# Incretin influence on type 2 diabetes mellitus remission after Roux-en-Y gastric bypass

**DOI:** 10.1590/0102-672020260000051e1980

**Published:** 2026-10-05

**Authors:** Flavio Masato Kawamoto, Roberto De-Cleva, Denis Pajecki, Daniel Riccioppo Cerqueira Ferreira Oliveira, Leila Antonangelo, Priscila Caproni, Marco Aurelio Santo

**Affiliations:** 1 Universidade de São Paulo, School of Medicine, University Hospital, Department of Gastroenterology – São Paulo (SP), Brazil.; 2 Universidade de São Paulo, School of Medicine, University Hospital, Central Laboratory – São Paulo (SP), Brazil.

**Keywords:** Bariatric Surgery, Diabetes Mellitus, Type 2, Incretins, Glucagon-Like Peptide 1, Ghrelin, Cirurgia Bariátrica, Diabetes Mellitus Tipo 2, Incretinas, Peptídeo 1 Semelhante ao Glucagon, Grelina

## Abstract

**Background:**

Roux-en-Y gastric bypass promotes weight loss in patients with severe obesity and improves type 2 diabetes mellitus, which, in some cases, can lead to remission. The action of enteric hormones is relevant for the initial outcomes and late disease evolution.

**Aims:**

To evaluate the influence of enteric hormone secretion on the evolution of type 2 diabetes mellitus three years after Roux-en-Y gastric bypass.

**Methods:**

A total of 20 patients were evaluated, divided into 2 groups: group 1, glycated hemoglobin<6%, and group 2, glycated hemoglobin≥6%. Hormone secretion (ghrelin, GLP-1, and GIP) was analyzed at pre-specified time points following a standard meal.

**Results:**

Group 1 had higher mean secretion at 30 and 120 minutes ([992.60±806.78 vs. 407.97±186.80, p=0.039, p<0.05] and [418.72±219.11 vs. 240.14±121.11, p=0.037, p<0.05], respectively) than group 2. Group 1 presented higher GLP values at 60 [724 (195–1,483) vs. 214 (67–424), p=0.038, p<0.05], 90 [473 (200–943) vs. 180 (120–318), p=0.016, p<0.05] and 120 minutes [565 (278–770) vs. 163 (81–339), p=0.008, p<0.05] compared to group 2. There was no difference between the groups regarding ghrelin.

**Conclusions:**

Higher secretion of GIP and GLP-1 was observed in patients with type 2 diabetes mellitus remission. The incretin factor is important in late disease evolution even in a group of patients with severe diabetes.

## ARTICLE HIGHLIGHTS

- Gastric bypass provides effective glycemic control in type 2 diabetes patients.
- Weight loss and type 2 diabetes mellitus control are due to the incretin effect and diet modification.
- Long-term incretin effect influences type 2 diabetes mellitus control.

## Summary

**CENTRAL MESSAGE:**

Bariatric surgery is the treatment of choice for individuals with severe obesity and type 2 diabetes mellitus. The return of blood glucose to normal levels in the first few days after surgery suggests that weight loss is not solely responsible for the patient's metabolic improvement. Effects related to improved stimulation and secretion of enteric hormones, especially glucagon-like peptide-1 (GLP-1) and peptide YY (PYY), with actions on the central nervous system and digestive tract, can be responsible for the early improvement of type 2 diabetes mellitus.

**PERSPECTIVES:**

Gastric bypass promotes weight loss and better type 2 diabetes mellitus control. These effects extend to the late postoperative period. However, elevated glycated hemoglobin levels have been identified as a marker of worse prognosis in the evolution of type 2 diabetes mellitus after the procedure. Enteric hormone secretion influences the control of type 2 diabetes mellitus. Higher glucagon-like peptide-1 (GLP-1) secretion levels were associated with better glycemic control.

## VISUAL ABSTRACT

**Figure:**
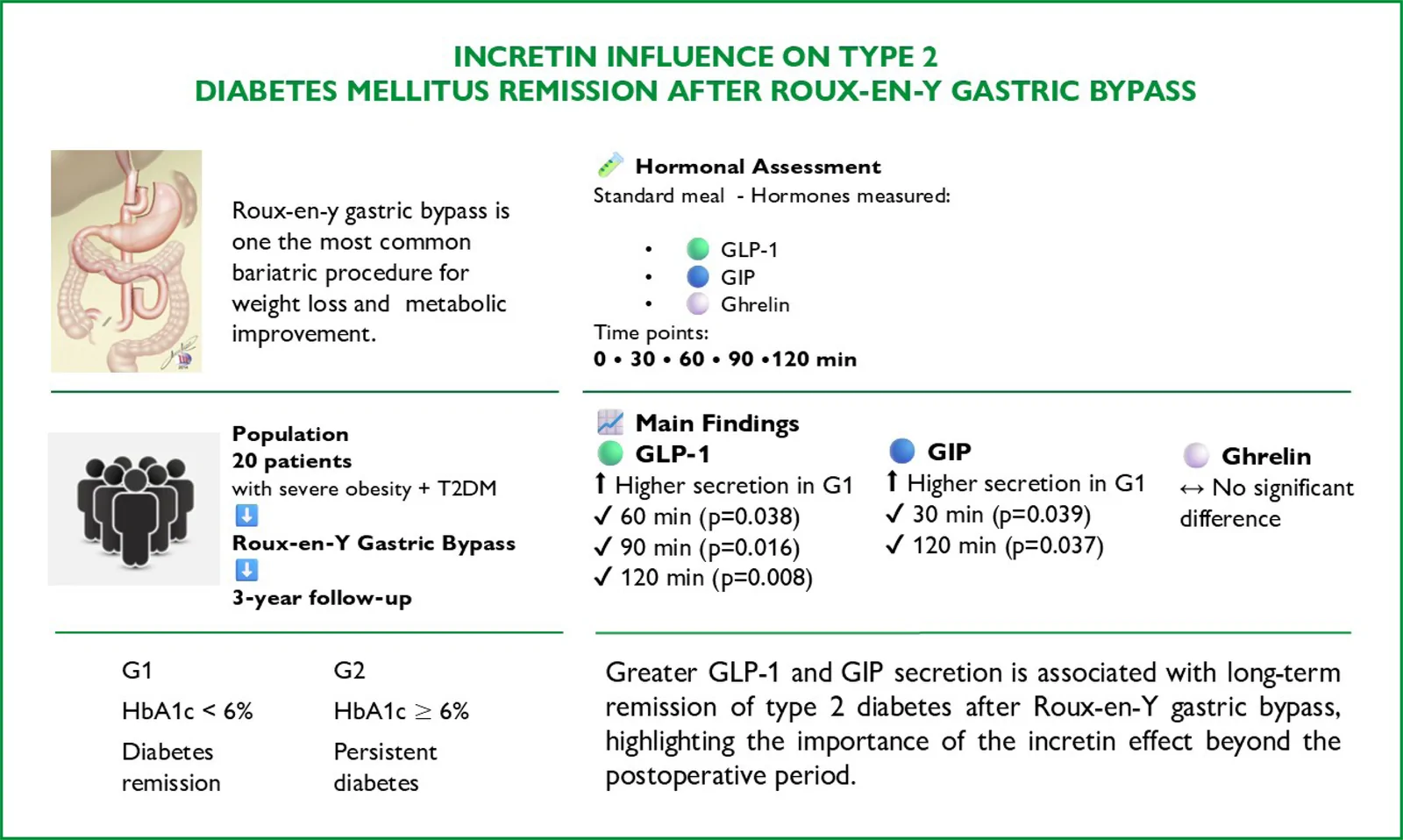

## RESUMO

**Racional:**

O bypass gástrico em Y de Roux promove perda ponderal em pacientes com obesidade grave e melhora do diabetes mellitus tipo 2, podendo, em alguns casos, levar à remissão da doença. A ação dos hormônios enteroendócrinos é relevante tanto nos resultados iniciais quanto na evolução tardia.

**Objetivos:**

O objetivo deste estudo foi avaliar a influência da secreção de hormônios enteroendócrinos ((grelina, GLP-1 e GIP) na evolução do diabetes mellitus tipo 2, após três anos da realização do bypass gástrico em Y de Roux.

**Métodos:**

Foram avaliados 20 pacientes submetidos ao bypass gástrico em Y de Roux, divididos em dois grupos: grupo 1 (G1), com hemoglobina glicada (HbA1c)<6%, e grupo 2 (G2), com HbA1c≥6%. A secreção hormonal (grelina, GLP-1 e GIP) foi avaliada em tempos previamente determinados após a ingestão de refeição padrão.

**Resultados:**

O G1 apresentou maiores médias de secreção hormonal aos 30 e 120 minutos, respectivamente, em comparação ao G2 [992,60±806,78 vs. 407,97±186,80; p=0,039, p<0,05] e [418,72±219,11 vs. 240,14±121,11; p=0,037, p<0,05]. Os valores de GLP-1 foram significativamente maiores no G1 aos 60 [724 (195–1.483) vs. 214 (67–424); p=0,038, p<0,05], 90 [473 (200–943) vs. 180 (120–318); p=0,016, p<0,05] e 120 minutos [565 (278–770) vs. 163 (81–339); p=0,008, p<0,05]. Não houve diferença estatisticamente significativa entre os grupos em relação à grelina.

**Conclusões:**

Pacientes com remissão do diabetes mellitus tipo 2 apresentaram maior secreção de GIP e GLP-1. O efeito incretínico mostrou-se relevante na evolução tardia, mesmo em pacientes com diabetes de maior gravidade.

## INTRODUCTION

Obesity is characterized by the accumulation of excessive fat associated with the development of comorbidities and a low-grade systemic inflammatory state^3,25,26^.

Bariatric surgery is the treatment of choice for individuals with severe obesity and type 2 diabetes mellitus (T2DM)^12,29,30^. The return of blood glucose to normal levels in the first few days after surgery suggests that weight loss is not solely responsible for the patient's metabolic improvement^10,32,36^. Effects related to improved stimulation and secretion of enteric hormones, especially glucagon-like peptide-1 (GLP-1) and peptide YY (PYY), with actions on the central nervous system and digestive tract, can be responsible for the early improvement of T2DM^27^.

The early incretin effect was demonstrated by Fernandes et al.^10^ in a clinical trial that observed higher GLP-1 secretion in patients undergoing Roux-en-Y gastric bypass (RYGB) compared with equivalent weight loss with diet alone^2,10^.

Even without weight loss, an improvement in glycemic metabolism is observed in patients undergoing RYGB or biliopancreatic diversion a few days after surgery^19^.

Some authors believe that the incretin effect is more important for diabetes improvement in the early postoperative period until at least two years^13^.

The present study aimed to evaluate the influence of the incretin effect on the late postoperative evolution of T2DM.

## METHODS

Twenty patients with severe obesity and T2DM who underwent RYGB at the Bariatric and Metabolic Surgery Unit of the Universidade de São Paulo University Hospital. All patients were included in the study after signing the Informed Consent Form. The study was approved by the Research Ethics Committee of Hospital das Clínicas of Universidade de São Paulo School of Medicine (CAAE No.: 16824813.2.0000.0068)

Patients were divided into two groups according to the American Diabetes Association (ADA) criteria^1^ for complete diabetes mellitus remission in the late postoperative period (three years):

- Group 1: glycated hemoglobin (HbA1c)<6%;
- Group 2: glycated hemoglobin (HbA1c)≥6%.

Enteric hormones (ghrelin, GLP-1, and GIP) were measured after a standardized meal ingested within 10 minutes (one Nutren^®^ 1.5 vial), consisting of a 200 mL solution with about 300 Kcal (11 g of protein, 44 g of carbohydrate, and 9.6 g of fat). The enteric hormone profile was collected at 30, 60, 90, and 120-minute time points. The analysis was carried out using Luminex^®^ xMAP technology, a multiplex method that quantifies several markers simultaneously.

### Statistical analysis

Categorical variables were presented in tables containing absolute and relative frequencies, and the association between them was compared with the χ^2^ test or Fisher's exact test. The Kolmogorov-Smirnov test was used to evaluate normal distribution. Quantitative variables were presented as mean and standard deviation and compared with the Student's t-test. For non-parametric variables, we described the median and interquartile range and compared distributions with the Mann-Whitney test.

Variables that showed association (p<0.10) in the univariate analysis were used in logistic regression models (with stepwise procedure for variable selection) to evaluate predictors of glucose, HbA1c, and Homeostatic Model Assessment for Insulin Resistance (HOMA-IR) index in the three-year postoperative period.

Values of p<0.05 were considered significant, and IBM-SPSS software (IBM SPSS Statistics for Windows, Version 19.0. Armonk, NY: IBM Corp) was used.

## RESULTS

A total of 20 patients were evaluated, most of whom were female (75%). Demographic and clinical variables are shown in Table 1. In the preoperative period, the weight was 116±16 kg, and the body mass index (BMI) was 43.5±5.6 kg/m^2^. All patients had T2DM with a median and interquartile disease duration of 10 (7–15) years. Twelve (60%) patients were using insulin (Table 1).

**Table 1:** Demographic and clinical characteristics of the study population.

Variable	n=20
Male sex, n (%)	5 (25)
Age (years), mean±SD	54±7
Age>50 years, n (%)	12 (60)
Weight (kg), mean±SD	116±16
Height (m), mean±SD	1.6±0.1
Body mass index (kg/m²), mean±SD	43.5±5.6
Duration of T2DM (years), median (IQR)	10 (7–15)
Insulin use, n (%)	12 (60)

T2DM: type 2 diabetes mellitus; IQR: interquartile range; SD: standard deviation.

There was a significant reduction (p=<0.001, p<0.05) in weight, BMI, glycemia, HbA1c, and HOMA-IR index from 6 months to 3 years compared with the preoperative period (Table 2).

**Table 2:** Weight and laboratory data during the 3-year follow-up.

(n=20)	Weight (Kg)	BMI (Kg/m^2^)	HbA1c (g/dL)	Glycemia (MG/DL)	HOMA-IR
Pre	116.0±15.8	43.5±5.6	8.8±2.1	187.2±79.6	16.8±19.7
6 months	87.8±14.0	35.4±5.5*	6.3±1.1*	118,7±34,4*	2.7±2.5*
1 year	79.5±12.4	31.4±3.5*	6.1±1.0*	117,1±45,6*	1.9±1.1*
2 years	79.3±12.9	31.1±3.9*	6.4±1.4*	112,8±37,3*	2.4±1.7*
3 years	80.2±15.2	31.1±4.7*	6.3±1.3*	109,4±27,8*	2.0±1.5*

* p<0.001 compared with the preoperative period (Analysis of variance for repeated measures); BMI: body mass index; HOMA-IR: Homeostatic Model Assessment for Insulin Resistance; HbA1c: glycated hemoglobin; Pre: preoperative period.

There was no association between preoperative demographic and clinical characteristics and HbA1c (Table 3).

**Table 3:** Preoperative demographic and clinical characteristics by whether type 2 diabetes mellitus remission happened during the three-year postoperative period.

Variable	1	2	p-value
	9 (45%)	11 (55%)	
Gender (male)	2 (22.2%)	3 (27.3%)	1.000^a^
Age (years)	55 (45–59)	55 (50–60)	0.673^b^
Age>50 years	5 (55.6%)	7 (63.6%)	1.000^a^
Height (m)	1.59±0.08	1.64±0.12	0.348^c^
Weight (kg)	115.67±15.03	116.18±17.17	0.945^c^
BMI (kg/m^2^)	45.21±6.12	40.85±3.74	0.066^c^
T2DM time (years), median, and IQR	10 (8–10)	10 (5–18)	0.641^b^
Insulin treatment	5 (55.6%)	7 (63.6%)	1.000^a^

a Fisher's exact test;

b Mann-Whitney test;

c Student's t-test; BMI: body mass index; T2DM: type 2 diabetes mellitus; IQR: interquartile range.

There was no association between weight, weight reduction, BMI and HbA1c in the three-year postoperative period. Significantly higher blood glucose and HbA1c were observed in group 2 compared with group 1, in the 6-month, 1, 2 and 3-year postoperative periods. The HOMA-IR index was not statistically significant between the groups.

The results of enteric hormones are shown in Table 4. Group 1 had a slightly higher mean GLP-1 secretion curve at 30 (p=0.039) and 120 minutes (p=0.037). Group 1 presented higher GLP-1 values at 60 (p=0.038), 90 (p=0.016), and 120 minutes (p=0.008, p<0.05) compared with group 2. There was no difference in ghrelin.

**Table 4:** GIP, GLP1, and ghrelin by whether type 2 diabetes mellitus remission happened during the three-year postoperative period.

Variable		HbA1c (3 years postoperatively)		p-value
		G1	G2	
		9 (45%)	11 (55%)	
GIP (min)				
	0	261.48±178.77	212.25±59.55	0.420^b^
	30	992.60±806.78	407.97±186.80	0.039^b^
	60	573.95±324.01	419.44±309.14	0.290^b^
	90	442.05±278.07	321.89±169.79	0.259^b^
	120	418.72±219.11	240.14±121.11	0.037^b^
GLP-1, median and IIQ (min)				
	0	286 (151–339)	153 (133–218)	0.140^a^
	30	1,719 (185–3,278)	247 (115–572)	0.096^a^
	60	724 (195–1,483)	214 (67–424)	0.038^a^
	90	473 (200–943)	180 (120–318)	0.016^a^
	120	565 (278–770)	163 (81–339)	0.008^a^
Ghrelin, median and IIQ (min)				
	0	340 (234–1,190)	688 (459–1,563)	0.226^a^
	30	225 (169–734)	919 (406–1,344)	0.151^a^
	60	228 (147–1,132)	1086 (510–1,456)	0.131^a^
	90	191 (171–618)	921 (462–1,555)	0.049^a^
	120	228 (177–1,019)	938 (806–1,375)	0.096^a^

a Mann-Whitney test;

b Student's t-test; G1: group 1; G2: group 2; GIP: glucose-dependent insulinotropic polypeptide; GLP-1: glucagon-like peptide-1; IIQ: interquartile interval; HbA1c: glycated hemoglobin.

For each 1-unit increase in HbA1c in the 6-month postoperative period, the risk of HbA1c>6% at 3-year follow-up increases 38.5-fold (Table 5).

**Table 5:** Multiple logistic regression model for HbA1c>6% at three years of follow-up.

Variable	Estimated parameter	Standard Error	OR	95%CI		p-value
HbA1c (%), 6 months postoperative	3.65	1.84	38.560	1.043	1,424.999	0.047
Constant	-21.55	10.64				

CI: confidence interval; OR: odds ratio; HbA1c: glycated hemoglobin.

## DISCUSSION

The beneficial effects of metabolic surgery on glycemic metabolism are well known^20,31,33,41^. Schauer et al.^33^, in a randomized study comparing patients undergoing surgery with patients receiving medical treatment, demonstrated a higher rate of weight loss and diabetes remission in the surgery group at a late 5-year follow-up.

In our study, at the 3-year follow-up, we observed significant improvement in the entire group in weight loss, BMI reduction, HbA1c reduction, fasting glucose, and HOMA-IR index.

No predictive preoperative factor was significant in identifying better T2DM outcomes after surgery. However, in the T2DM control analysis by multiple logistic regression, it was observed that up to 6 months, the worst glycemic control of some patients is less likely to achieve control with an A1c level below 6%, reflecting the severity of T2DM in these patients.

This study has some limitations related to its small sample size. Despite the homogeneity among the participants, most were using insulin because of the limited availability of other oral hypoglycemic agents in public health care.

Weight loss was similar at all time points analyzed and was not a significant factor for better glycemic control. Some studies have shown that increased GLP-1 secretion after gastric bypass promoted better beta cell function^16,18,24,34^.

GLP-1 is a hormone produced by L cells in the ileum and colon after meals, and influences glycemic control^9,15,35^. Increased GLP-1 secretion in the postprandial period stimulates the release of insulin. This process is an additional factor that contributes to the improvement of T2DM in the postoperative period of gastric bypass, as the surgical procedure intensifies the hormonal response after meals, helping regulate blood glucose levels. The impact of GLP-1, both with analogues and by the incretin phenomenon observed in the postoperative period, has been observed in several studies^7,17,40^. In this context, the LEADER trial^19^ highlighted that, among 9,340 diabetic patients, the use of GLP-1 not only improved glycemic control but also provided significant cardiovascular protection benefits^4^.

The study by Umeda et al.^36^ showed an improvement in glycemic control in the early phase after RYGB. In the long term, this improvement in glycemic control is maintained, influenced by additional factors such as pancreatic reserve, disease duration, and medication control. In addition, other studies confirmed the continuity of the incretin effect, contributing to the maintenance of positive outcomes^5,8^.

Significantly higher GLP-1 secretion was observed in group 1 at all measurements compared to group 2, with statistically significant differences at 60, 90, and 120 minutes (p<0.05).

GIP is a hormone produced by K cells in the small intestine and induces insulin secretion after food intake. An experimental study with knockout mice lacking the GIP receptor showed glucose intolerance after oral administration of glucose. Together with GLP-1, GIP is considered the main contributor to the incretin response^11,21,23^, as demonstrated in studies using mice with a knockout for both GLP-1 and GIP, which show an additive effect^37^.

In our study, we observed that the mean GIP secretion was significantly higher in group 1 at all dosages, remaining high until the end of the experiment, as observed with GLP-1.

Ghrelin is a hormone that plays an important role in appetite regulation and body weight control. It is directly associated with weight gain through activation of neuronal pathways in the hypothalamus, the hunger control center of the brain. When ghrelin is released, it stimulates these pathways, increasing feelings of hunger and encouraging food intake^6,14,22,39^.

In addition to its impact on appetite and weight management, ghrelin also influences glycemic metabolism. It interferes with the regulation of blood glucose levels by reducing insulin secretion, the hormone responsible for allowing cells to use glucose. This reduction in insulin secretion may lead to less effective control of glucose levels. These effects of ghrelin have been proven in several studies, both in animal and human models, evidencing its multifaceted role in the regulation of metabolism and appetite^28^.

In our study, we found that ghrelin secretion was higher in the group of patients with worse glycemic control (group 2) only at 90 min, with significance, supporting the hypothesis that ghrelin has a diabetogenic effect. However, despite this association, we cannot attribute the worse glycemic control observed in this group exclusively to ghrelin, since, by itself, it did not demonstrate this isolated potential.

The incretin effect, highlighted in several previous studies^13,19,38^, has shown a significant impact in the early stages of T2DM treatment. In our study, we found that this effect not only manifests initially, but persists over time, continuing to exert a significant influence on glycemic control even after three years.

This prolonged influence is particularly evident in GLP-1 and GIP action, which are important for T2DM control. GLP-1, for example, stimulates insulin secretion in response to dietary intake and improves insulin sensitivity, while GIP contributes to increased insulin response. Together, these hormones help optimize glycemic metabolism, highlighting their importance not only in the period immediately after medical intervention, but also in their sustained long-term impact on T2DM management.

## CONCLUSIONS

Gastric bypass is effective for weight loss and improved T2DM control. These effects extend to the late postoperative period. However, elevated HbA1c levels have been identified as a marker of worse prognosis in the evolution of T2DM after the procedure.

Enteric hormone secretion influences the control of T2DM. Higher GLP-1 secretion levels are associated with better glycemic control.

## Data Availability

The datasets generated and/or analyzed during the current study are available from the corresponding author upon reasonable request.
